# Supplementary material for: RNA binding protein IGF2BP2 expression is induced by stress in the heart and mediates dilated cardiomyopathy
Source: Commun Biol. 2023 Dec 5;6:1229. doi: 10.1038/s42003-023-05547-x (PMC10698010; doi:10.1038/s42003-023-05547-x)
Supplement: Supplementary file 2 — Supplementary Information [file 42003_2023_5547_MOESM2_ESM.pdf]

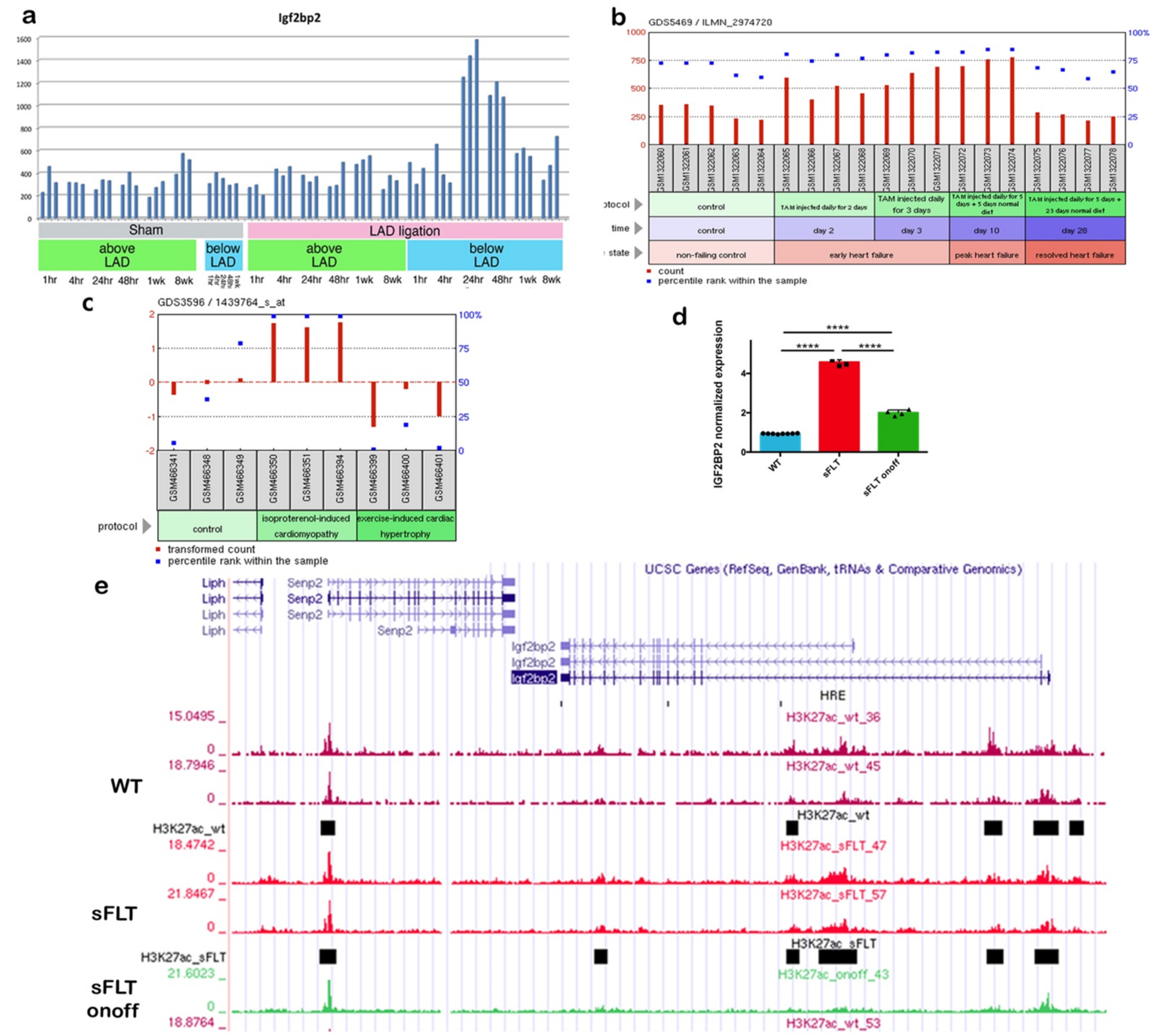

Supplementary Fig. 1. Cardiac stress models and IGF2BP2 expression
(a) IGF2BP2 mRNA expression was assayed, from 1 hour to 8 weeks, in Sham-operated and LAD ligated mice at levels above (unaffected area) and below (affected area) of the LAD (GEO data set GDS488). (b) Microarray data of IGF2BP2 expression from early (2,3 days), late (10 days), and resolved (28 days) heart failure in a MerCreMer mouse model (GEO data set GDS5469). IGF2BP2 mRNA expression is upregulated already at early stages and then down regulated upon recovery. (c) Microarray data of IGF2BP2 expression in isoproterenol-induced cardiomyopathy compared to exercised induced cardiac hypertrophy (GEO data set GDS3596). IGF2BP2 mRNA expression is upregulated in isoproterenol-induced cardiomyopathy and unchanged in exercise induced cardiac hypertrophy. (d) IGF2BP2 RNA expression in hearts from wildtype (WT), sFlt upregulated (sFLT), and sFlt upregulated and then downregulated (sFLT onoff) was compared on a gene array (p<0.0001). (e) ChIP analysis of H3K27ac - enhancer marks in the genomic region of IGF2BP2 in hearts isolated from WT, sFLT upregulated, and sFLT upregulated and then downregulated mice. Black boxes represent significant peaks. Genome assembly: GRCm38/mm10. ****p<0.0001. Error bars indicate standard error of mean.

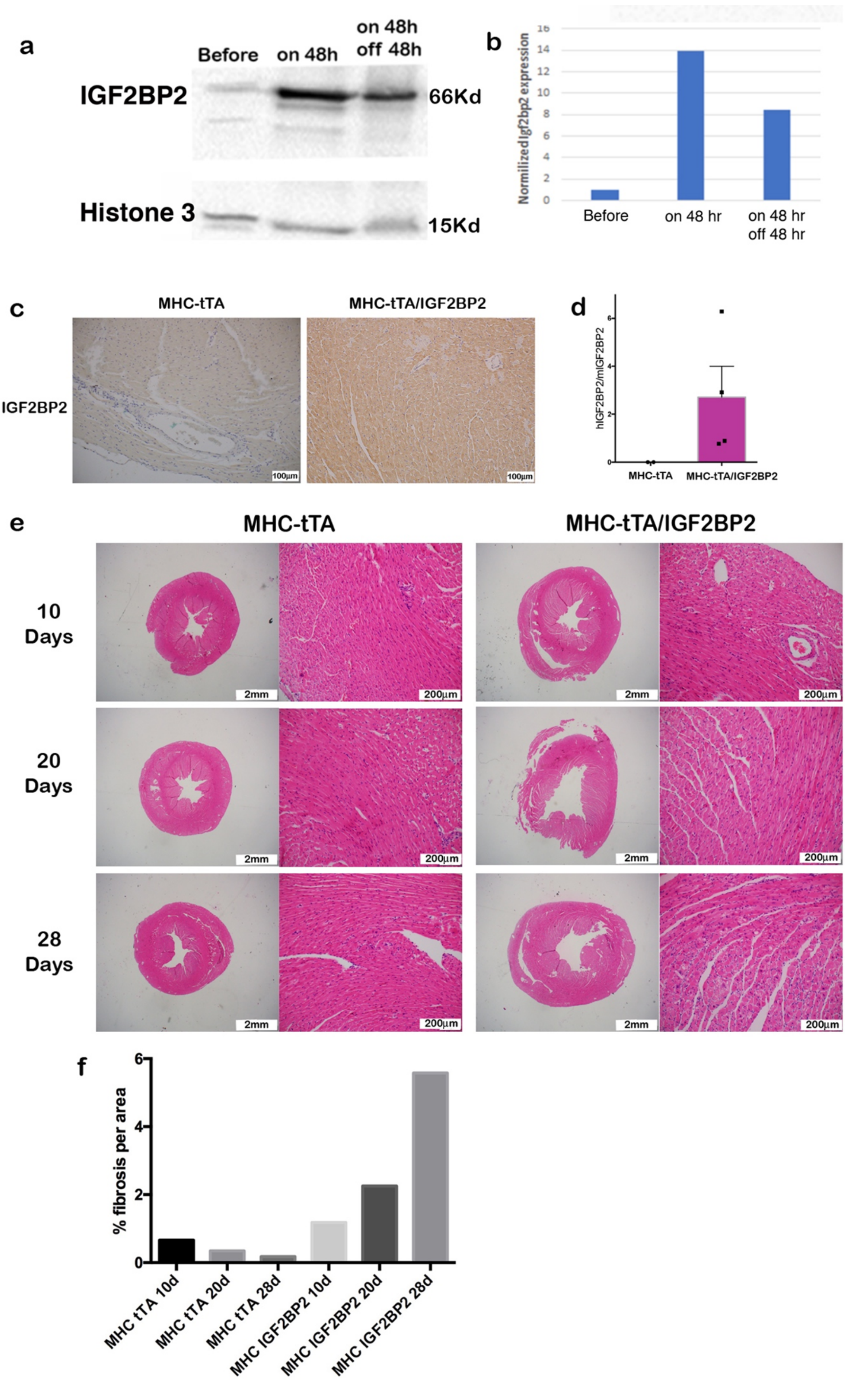
a
Before
on 48h
on 48h
off 48h
IGF2BP2
66Kd
Histone 3
15Kd
b
Normilized Igf2bp2 expression
Before
on 48 hr
on 48 hr
off 48 hr
c
MHC-tTA
MHC-tTA/IGF2BP2
IGF2BP2
100μm
d
hIGF2BP2/mIGF2BP2
MHC-tTA
MHC-tTA/IGF2BP2
e
MHC-tTA
MHC-tTA/IGF2BP2
10
Days
20
Days
28
Days
2mm
200μm
f
% fibrosis per area
MHC tTA 10d
MHC tTA 20d
MHC tTA 28d
MHC IGF2BP2 10d
MHC IGF2BP2 20d
MHC IGF2BP2 28d

Supplementary Fig. 2. Expression of IGF2BP2 in transgenic mice and histology of the hearts
(a) Protein extracts from hearts from MHC-tTA/IGF2BP2 mice maintained on tetracycline all the time (before), fed water with tetracycline for 48 hours (on 48h), or fed water with tetracycline for 48 hours and then replaced with regular water for 48 hours (on 48h, off 48h) were electrophoresed and then analyzed for expression of IGF2BP2. Histone 3 was used as a loading control. Samples were run on the same gel. (b) Quantification of IGF2BP2 levels from (a), normalized to Histone 3. (c) Hearts from MHC-tTA and MHC-tTA/IGF2BP2 mice were stained for IGF2BP2 expression with an anti-IGF2BP2 antibody. Scale bar is 100µm. (d) The level of transgene expression (normalized to endogenous expression; hIGF2BP2/mIGF2BP2) in primary cardiomyocytes was measured by RT-PCR 5 days after transgene induction (withdrawal from tetracycline). Each bar represents 4 biological repeats ± SEM. (e) H&E staining of MHC-tTA and MHC-tTA/IGF2BP2 hearts at the time points indicated after the beginning of transgene induction (in mice 8-10 weeks old). Scale bar is 1mm and 100µm in the higher magnification (right panels). f. Analysis of the levels of fibrosis in the sections shown in Fig. 2h.

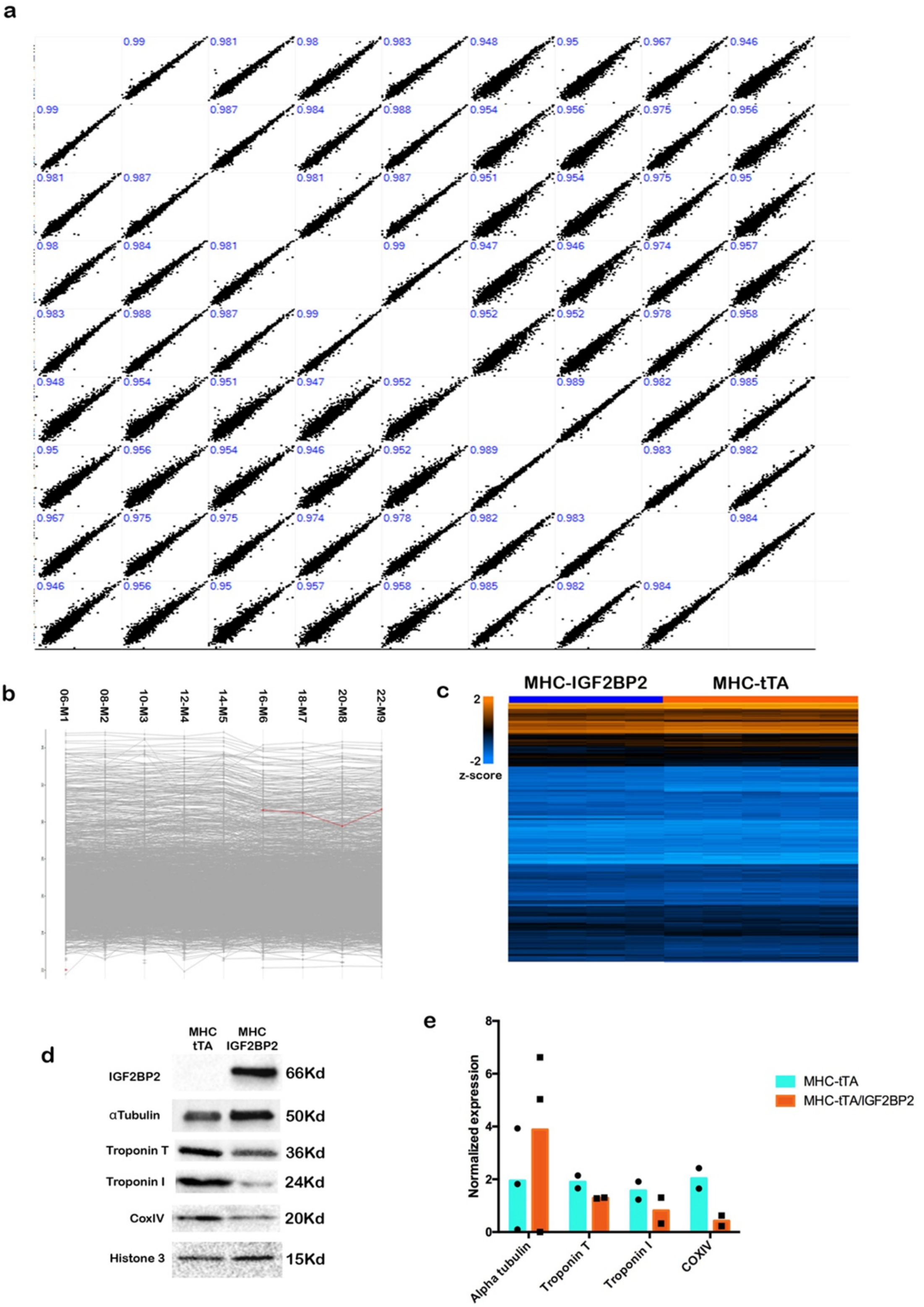

a
0.99 0.981 0.98 0.983 0.948 0.95 0.967 0.946
0.99 0.987 0.984 0.988 0.954 0.956 0.975 0.956
0.981 0.987 0.981 0.987 0.951 0.954 0.975 0.95
0.98 0.984 0.981 0.99 0.947 0.946 0.974 0.957
0.983 0.988 0.987 0.99 0.952 0.952 0.978 0.958
0.948 0.954 0.951 0.947 0.952 0.989 0.982 0.985
0.95 0.956 0.954 0.946 0.952 0.989 0.983 0.982
0.967 0.975 0.975 0.974 0.978 0.982 0.983 0.984
0.946 0.956 0.95 0.957 0.958 0.985 0.982 0.984
b
06-M1
08-M2
10-M3
12-M4
14-M5
16-M6
18-M7
20-M8
22-M9
c
MHC-IGF2BP2
MHC-tTA
2
-2
z-score
d
MHC tTA
MHC IGF2BP2
IGF2BP2 66Kd
αTubulin 50Kd
Troponin T 36Kd
Troponin I 24Kd
CoxIV 20Kd
Histone 3 15Kd
e
Normalized expression
8
6
4
2
0
Alpha tubulin
Troponin T
Troponin I
COXIV
MHC-tTA
MHC-tTA/IGF2BP2

Supplementary Fig. 3. Data from Mass Spectrometry analysis
(a) A multi scatter plot with Pearson correlations of all 9 samples described in figure 5. (b) IGF2BP2 profile plot in all 9 samples. (c) Unsupervised hierarchical clustering of all 9 samples without Z score. Orange header, MHC tTA samples; Blue header, MHC IGF2BP2 samples. (d) Western blot validation of proteins shown to be up- (IGF2BP2) or down-regulated (Tubulin, Troponin T, Troponin I, and COXIV) by MS analysis mass spectrometry. Histone 3 was used as a loading control. Tubulin, Troponin T, and CoxIV blots were from different lanes on the same gel. Troponin I and Histone 3 were from different lanes on a separate gel. IGF2BP2 was run on a separate gel as well. (e) Quantification of the western blot analysis with two biological samples in each group ±SEM.

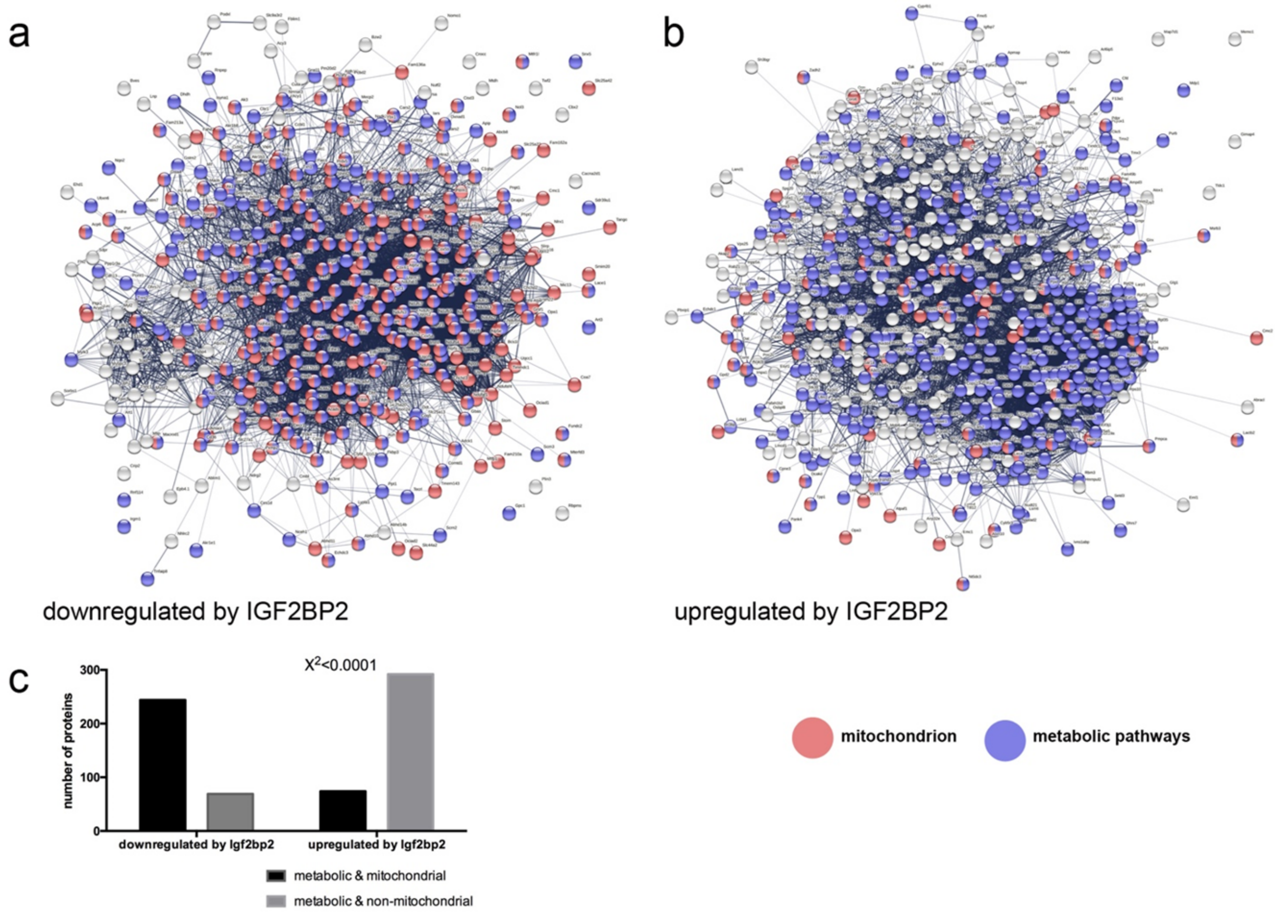

Supplementary Fig. 4. Protein-protein interaction network comparison of proteins down- or up-regulated by IGF2BP2 overexpression.

Proteins significantly down- (a) or up- (b) regulated (FDR=0.05; S=0.1) in the MS analysis (Supplemental File 1) were displayed in a Protein-Protein interaction analysis using the STRING website. Genes associated with the Mitochondrion Cellular Components gene set (GO:0005739) were colored pink and those associated with Metabolic Pathways Biological Processes (GO:0008152) were colored blue. A highly significant overlap (χ^2<0.0001) of metabolic and mitochondrial proteins is present in the downregulated as opposed to upregulated proteins (c).

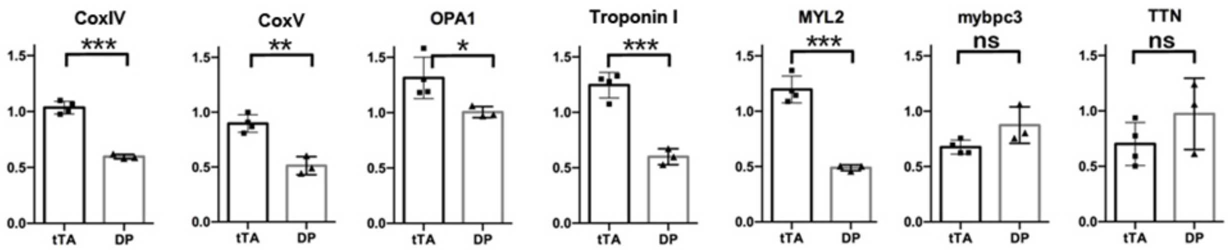

Supplementary Fig. 5. Real time PCR analysis of RNAs encoding proteins downregulated in hIGF2BP2-expressing mice.

The relative abundance of 7 RNAs encoding proteins downregulated in the proteomics experiment shown in Fig.5 and Supplementary Fig. 3 was assayed by real time PCR. Three or four biological repeats, with three technical repeats for each biological repeat, were performed for each gene. *, $p<0.05$; **, $p<0.01$; ***, $p<0.001$; ns, not significant. Error bars indicate standard error of mean.

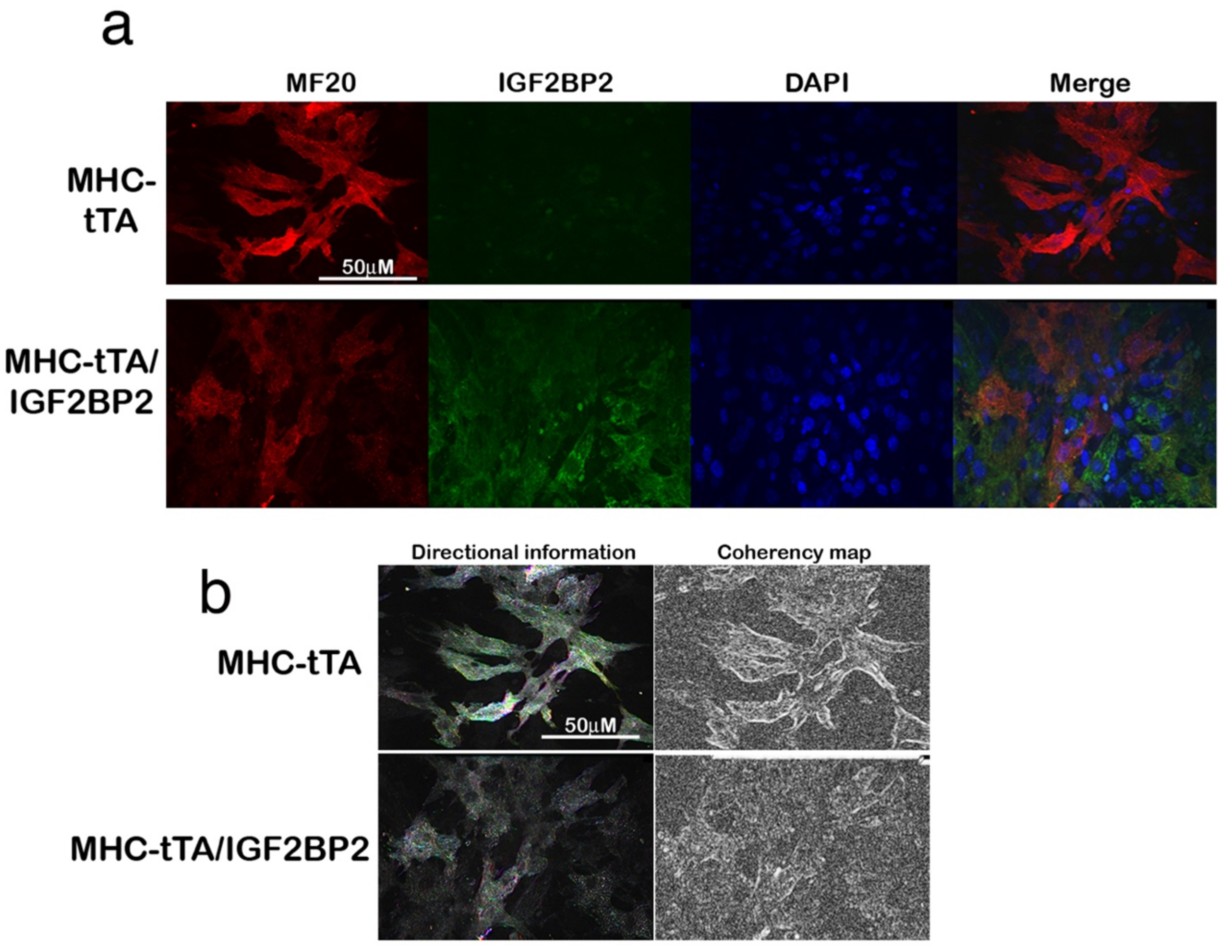

Supplementary Fig. 6. IGF2BP2 expression in primary cardiomyocytes affects sarcomere structure
(a) Primary cardiomyocytes isolated from the hearts of either MHC-tTA or MHC-tTA/IGF2BP2 mice were grown for 7 days in culture in the absence of tetracycline (to activate the transgene) and then stained for MF20, IGF2BP2 and DAPI. (b) Analysis using ImageJ OrientationJ plugin of directionality (left) and coherency (right) of the pixels of MF20 staining (346 MHC-tTA cells and 216 MHC-tTA/IGF2BP2 cells were counted).

Supplementary Figure 7 – Uncropped gels/blots

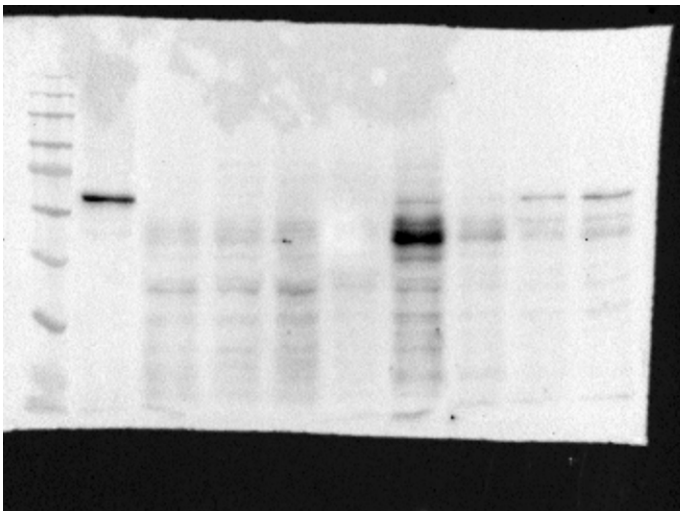

IGF2BP1

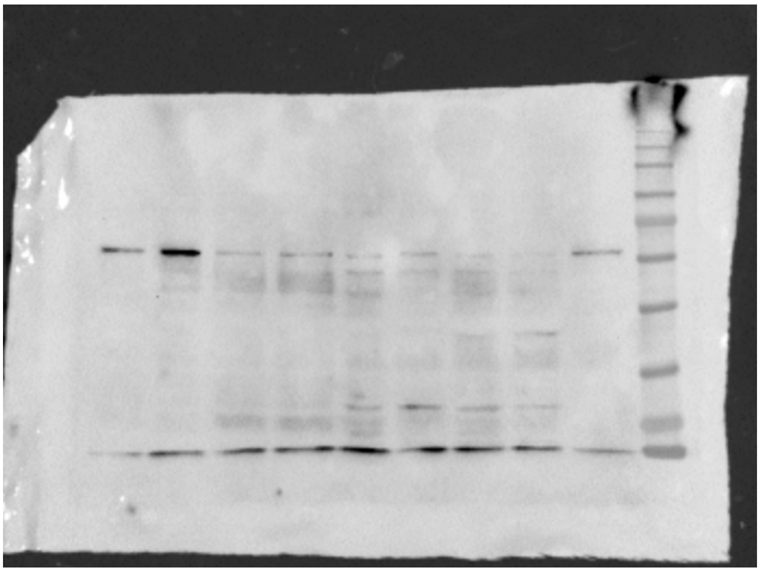

IGF2BP2

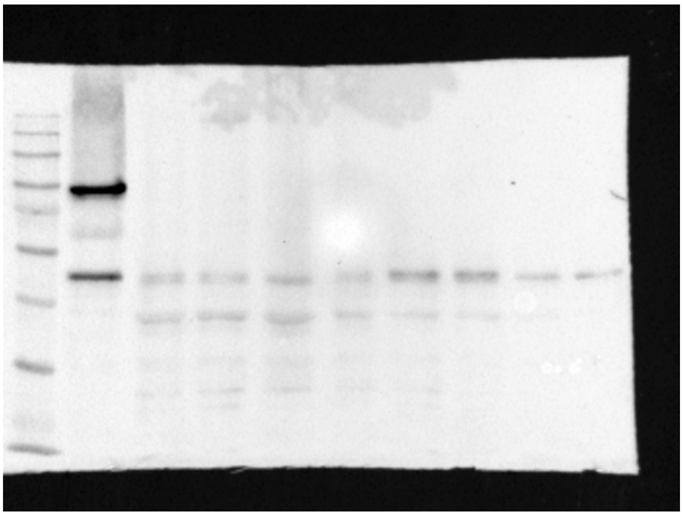
IGF2BP3

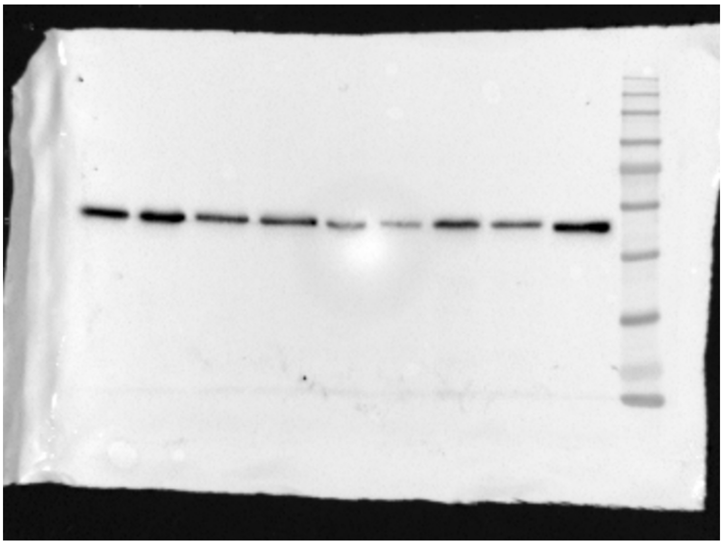
Tubulin

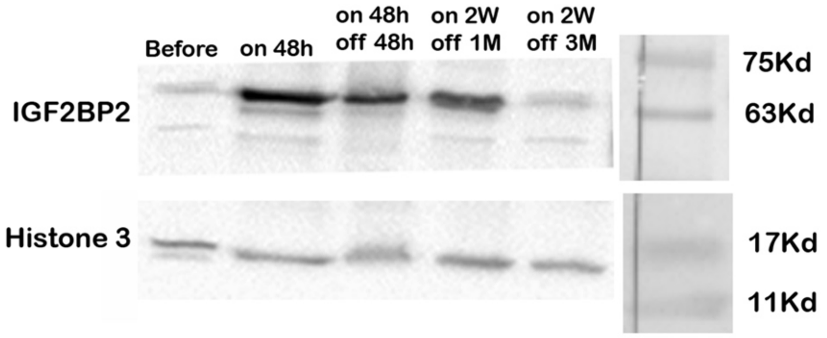

The membrane strips shown here were taken from the same lanes and separately incubated with antibody to the indicated protein.

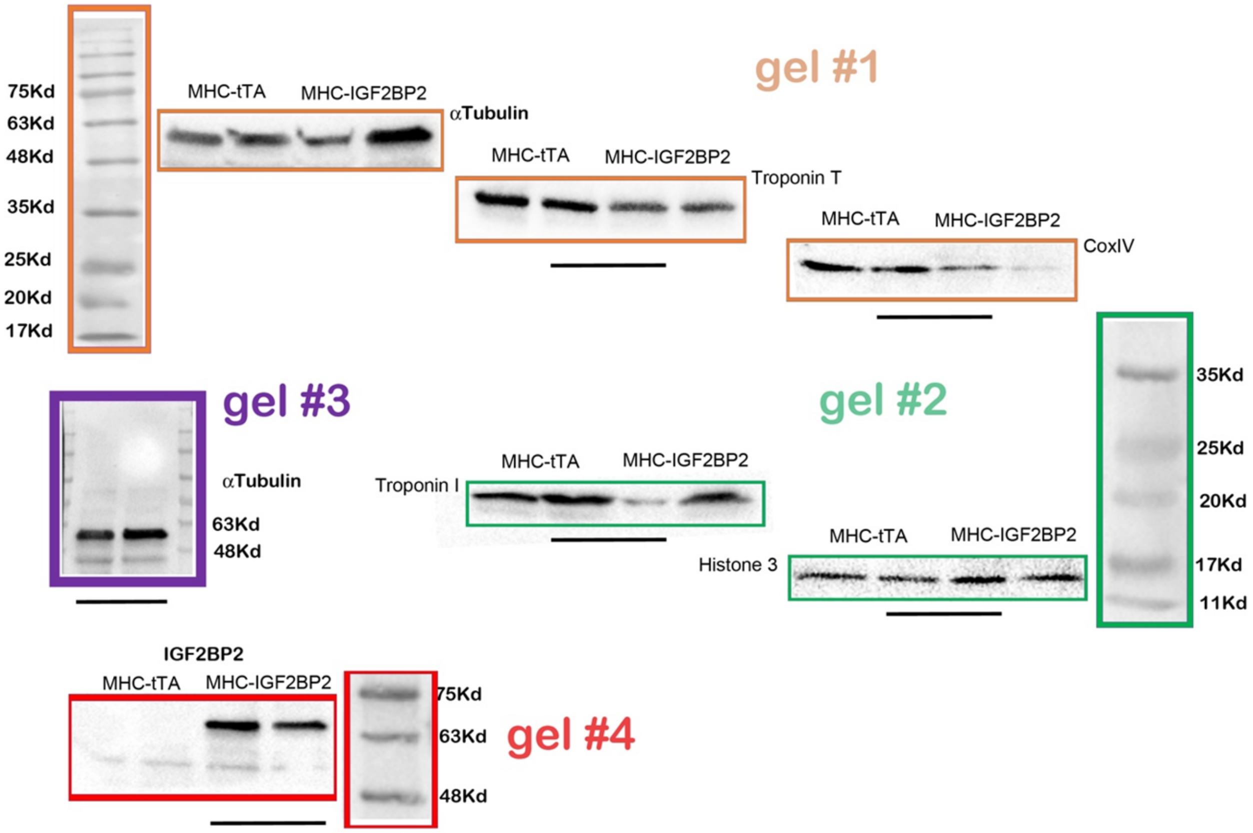

Protein extract from MHC-tTA or MHC-IGF2BP2 was loaded on adjacent lanes as shown above along with a protein ladder lane. The gels were electrophoresed and then transferred to a membrane. In order to conserve on antibodies, the membrane was cut horizontally at different positions along the running axis, based on the position of the ladder, to include adjacent lanes of either four samples (2 MHC-tTA, 2MHC-IGF2BP2, in gels #1,2, and 4) or 2 samples (1 MHC-tTA, 1 MHC-IGF2BP2 in gel #3) and then incubated with antibody recognizing the indicated protein. For ease of clarification in the figure above, membrane sections taken from the same gel are framed in the same color. Each membrane section was taken from a different set of lanes. The protein ladder that was run on the gel, but separated at the time the membrane was cut, is shown next to the membrane sections in its approximate position. Thus, the molecular weights indicated in Supplementary Fig. 3 are inferred from the antibody manufacturer.

Supplementary Fig. 3d was constructed from the underlined lanes in gels #1-4:
The Troponin T and CoxIV membrane sections were different groups of lanes, both taken from gel #1.
The Troponin I and Histone 3 membrane sections were different groups of lanes, both taken from gel #2.
The αTubulin membrane section was taken from gel #3.
The IGF2BP2 membrane section was taken from gel #4.

Supplementary Fig. 3e was calculated by scanning all of the lanes shown above.

Supplementary Table 1. Details of antibodies used in the paper

Primary ab	Source				
Protein	Rabbit	Dilution western	Dilution ICF	Company	Cat no.
IGF2BP2	Rabbit	1:3000	1:250	Eng Tan	
α/β Tubulin	Mouse	1:3000		Cell Signaling	2148s
α Tubulin	Mouse	1:1000		Cell Signaling	3873
Troponin T	Mouse	1:80		Hybridoma bank	
Troponin I	Mouse	1:80		Hybridoma bank	
MF20	Mouse	1:400	1:150	Hybridoma bank	
COXIV	Mouse	1:10,000		abcam	ab33985
Histone 3	Rabbit	1:1000		Cell Signaling	9701
α Actinin				Cell Signaling	6487s
Secondary ab					
DAPI	Goat anti Mouse		1:1000	Biorad	
488	Donkey anti Rabbit		1:400	Jackson immuno research	
Cy3			1:400	Jackson immuno research	

Supplementary Table 2. Primers used for real time PCR

Gene name	Forward primer	Reverse primer
COX1 mitochondrial DNA	GCAGGAGCATCACTAGACCTAAC	GGAGTTTGATACTGTGTTATGGCTGG
Chromosome 12 DNA	TTAGTCCGCAAAACCCAATC	CCATAAGCCAGAAGCAATCAC
ACTA1	GATTGACTCGTTTTACCTCATTTTGT	CTTTAATGCTTCAAGTTTTCCATTTC
Actn2	CCCTGGACTACACTGCCTTCT	AACAGCCTATACTTCAGCCTTTATTG
ANP	TCCTAAGCCCTTGTGGTGTG	TCGTGATAGATGAAGGCAGGA
COXB6	GGATGACCGCATAGCTGAA	GAGAAAGACAAAGAACAGAGGAGAG
IGF2BP2 mouse	CCTAGCCGTTTCTTTGTTGTG	CCCACAGGCCACATTTCT
IGF2BP2 human CDS	GGGAGGTGTTGGATGGACTT	GGCGGTTTCTGTGTCTGTGT
NDUFA1	AGTAACGGTGCGGAGATGTG	CGTTGGTGAATTTGTGGATGT
NDUFA8	TGGACCGTGTAGAGATGGGT	AAGAACACGAGATCGGCACG
HPRT	CAGTCCCAGCGTCGTGATTA	GGCCTCCCATCTCCTTCATG
