# Supplementary material for: RNA binding protein IGF2BP2 expression is induced by stress in the heart and mediates dilated cardiomyopathy
Source: Commun Biol. 2023 Dec 5;6:1229. doi: 10.1038/s42003-023-05547-x (PMC10698010; doi:10.1038/s42003-023-05547-x)
Supplement: Supplementary file 3 — Description of Additional Supplementary Files [file 42003_2023_5547_MOESM3_ESM.pdf]

Description of Additional Supplementary Files

File name: Supplementary Data 1
Description: Mass Spectrometry Data

File name: Supplementary Data 2
Description: The source data behind the graphs in the paper.
