# Supplementary material for: RNA binding protein IGF2BP2 expression is induced by stress in the heart and mediates dilated cardiomyopathy
Source: Commun Biol. 2023 Dec 5;6:1229. doi: 10.1038/s42003-023-05547-x (PMC10698010; doi:10.1038/s42003-023-05547-x)
Supplement: Supplementary file 6 — Reporting Summary [file 42003_2023_5547_MOESM6_ESM.pdf]

nature portfolio

Corresponding author(s): Joel Yisraeli

Last updated by author(s): Sept. 6, 2023

Reporting Summary

Nature Portfolio wishes to improve the reproducibility of the work that we publish. This form provides structure for consistency and transparency in reporting. For further information on Nature Portfolio policies, see our Editorial Policies and the Editorial Policy Checklist.

Statistics

For all statistical analyses, confirm that the following items are present in the figure legend, table legend, main text, or Methods section.

n/a	Confirmed	
☐	☒	The exact sample size (n) for each experimental group/condition, given as a discrete number and unit of measurement
☐	☒	A statement on whether measurements were taken from distinct samples or whether the same sample was measured repeatedly
☐	☒	The statistical test(s) used AND whether they are one- or two-sided<br>*Only common tests should be described solely by name; describe more complex techniques in the Methods section.*
☒	☐	A description of all covariates tested
☒	☐	A description of any assumptions or corrections, such as tests of normality and adjustment for multiple comparisons
☒	☐	A full description of the statistical parameters including central tendency (e.g. means) or other basic estimates (e.g. regression coefficient) AND variation (e.g. standard deviation) or associated estimates of uncertainty (e.g. confidence intervals)
☒	☐	For null hypothesis testing, the test statistic (e.g. F, t, r) with confidence intervals, effect sizes, degrees of freedom and P value noted<br>*Give P values as exact values whenever suitable.*
☒	☐	For Bayesian analysis, information on the choice of priors and Markov chain Monte Carlo settings
☒	☐	For hierarchical and complex designs, identification of the appropriate level for tests and full reporting of outcomes
☐	☒	Estimates of effect sizes (e.g. Cohen's d, Pearson's r), indicating how they were calculated

Our web collection on statistics for biologists contains articles on many of the points above.

Software and code

Policy information about availability of computer code

Data collection	No code was used to collect data
Data analysis	Graphpad Prism 6 was used for statistical analysis. MaxQuant (MQ) version 1.6.5.0 was used for analyzing raw data from the mass spectrometry. The bioinformatics was performed with Perseus suite (version 1.6.2.3)

For manuscripts utilizing custom algorithms or software that are central to the research but not yet described in published literature, software must be made available to editors and reviewers. We strongly encourage code deposition in a community repository (e.g. GitHub). See the Nature Portfolio guidelines for submitting code & software for further information.

Data

Policy information about availability of data

All manuscripts must include a data availability statement. This statement should provide the following information, where applicable:

- Accession codes, unique identifiers, or web links for publicly available datasets
- A description of any restrictions on data availability
- For clinical datasets or third party data, please ensure that the statement adheres to our policy

The mass spectrometry proteomics data have been deposited in the ProteomeXchange Consortium via the PRIDE partner repository with the dataset identifier PXD037623.

Human research participants

Policy information about studies involving human research participants and Sex and Gender in Research.

Reporting on sex and gender	n/a
Population characteristics	n/a
Recruitment	n/a
Ethics oversight	Biopsy samples used in this project were in accordance with the guidelines of the Medical University Innsbruck, Austria ethics commission under the authorization request: EK number 4077. BRISQ Tier 1 guidelines are reported in text.

Note that full information on the approval of the study protocol must also be provided in the manuscript.

Field-specific reporting

Please select the one below that is the best fit for your research. If you are not sure, read the appropriate sections before making your selection.

☒ Life sciences ☐ Behavioural & social sciences ☐ Ecological, evolutionary & environmental sciences

For a reference copy of the document with all sections, see nature.com/documents/nr-reporting-summary-flat.pdf

Life sciences study design

All studies must disclose on these points even when the disclosure is negative.

Sample size	Sample size was determined based on the expected effect size, expected variation, sample availability, and resource constraints. Due to the use of genetically homogeneous mouse samples, the biological variation between replicates was minimized, and largely influenced by experimental technique, and instrument limitations. As such, a sample size of 5-8 mice per group was used for most experiments.
Data exclusions	No data was excluded from the analysis.
Replication	Most experiments consisted of 3 biological repeats. All qPCR samples were run with at least 3 technical repeats. Each finding was supported by multiple experiments to ensure reproducibility of the same results. The mass spectrometry data consisted of 9 biological repeats, 4 experimental and 5 controls, all analyzed in parallel at the same time.
Randomization	Samples were taken from different litters and randomly allocated based on the experiment.
Blinding	Mice were identified by a unique alpha-numeric code with no genotype identifier to ensure blinding. After data collection was complete, samples were identified and allocated to the appropriate genotyping group to enable statistical analysis, as required.

Reporting for specific materials, systems and methods

We require information from authors about some types of materials, experimental systems and methods used in many studies. Here, indicate whether each material, system or method listed is relevant to your study. If you are not sure if a list item applies to your research, read the appropriate section before selecting a response.

Materials & experimental systems

n/a	Involved in the study	
☐	☒	Antibodies
☒	☐	Eukaryotic cell lines
☒	☐	Palaeontology and archaeology
☐	☒	Animals and other organisms
☒	☐	Clinical data
☒	☐	Dual use research of concern

Methods

n/a	Involved in the study	
☒	☐	ChIP-seq
☒	☐	Flow cytometry
☒	☐	MRI-based neuroimaging

Antibodies

Antibodies used	IGF2BP2 Rabbit 1:3000 1:250 Sonja Kessler<br>α/β Tubulin Mouse 1:3000 Cell Signaling 2148s

α Tubulin Mouse 1:1000 Cell Signaling 3873
Troponin T Mouse 1:80 Hybridoma bank
Troponin I Mouse 1:80 Hybridoma bank
MF20 Mouse 1:400 1:150 Hybridoma bank
COXIV Mouse 1:10,000 abcam ab33985
Histone 3 Rabbit 1:1000 Cell Signaling 9701
α Actinin Cell Signaling 6487s

Secondary ab
DAPI Goat anti Mouse 1:1000 Biorad
488 Donkey anti Rabbit 1:400 Jackson immuno research
Cy3 1:400 Jackson immuno research

Validation	All commercial antibodies were validated on the manufacturer's website. The Igf2bp2 antibody from Sonja Kessler was extensively validated by us.

Animals and other research organisms

Policy information about studies involving animals; ARRIVE guidelines recommended for reporting animal research, and Sex and Gender in Research

Laboratory animals	The TRE-human p62 (IGF2BP2) mice were on a DBA/2 background and have been previously described. Ages of the mice are described in text for each experiment.
Wild animals	The study did not involve wild animals.
Reporting on sex	The sex of the animals in the experiments is indicated in the text.
Field-collected samples	The study did not involve animals from the field.
Ethics oversight	All animal procedures were performed in accordance with the Hebrew University Faculty of Medicine animal ethics committee (MD-17-15389, MD-20-16134).

Note that full information on the approval of the study protocol must also be provided in the manuscript.
